# Screening for Viruses in Indigenous Greek Black Pigs

**DOI:** 10.3390/microorganisms12020315

**Published:** 2024-02-02

**Authors:** Hina Jhelum, Vasileios Papatsiros, Georgios Papakonstantinou, Ludwig Krabben, Benedikt Kaufer, Joachim Denner

**Affiliations:** 1 Institute of Virology, Free University Berlin, 14163 Berlin, Germany; hina.jhelum@fu-berlin.de (H.J.); ludwig.krabben@fu-berlin.de (L.K.); benedikt.kaufer@fu-berlin.de (B.K.); 2 Faculty of Veterinary Medicine, Clinic of Medicine (Farm Animal Medicine), University of Thessaly, 43100 Karditsa, Greece; vpapatsiros@vet.uth.gr (V.P.); geopapak@uth.gr (G.P.)

**Keywords:** xenotransplantation, virus safety, porcine cytomegalovirus/porcine roseolovirus (PCMV/PRV), hepatitis E virus, porcine endogenous retroviruses (PERVs)

## Abstract

The successful advancement of xenotransplantation has led to the development of highly sensitive detection systems for the screening of potentially zoonotic viruses in donor pigs and preventing their transmission to the recipient. To validate these methods, genetically modified pigs generated for xenotransplantation, numerous minipigs and other pig breeds have been tested, thereby increasing our knowledge concerning the pig virome and the distribution of pig viruses. Of particular importance are the porcine cytomegalovirus, a porcine roseolovirus (PCMV/PRV) and the hepatitis E virus genotype 3 (HEV3). PCMV/PRV has been shown to reduce the survival time of pig transplants in non-human primates and was also transmitted in the first pig heart transplantation to a human patient. The main aim of this study was to determine the sensitivities of our methods to detect PCMV/PRV, HEV3, porcine lymphotropic herpesvirus-1 (PLHV-1), PLHV-2, PLHV-3, porcine circovirus 2 (PCV2), PCV3, PCV4 and porcine parvovirus 1 (PPV1) and to apply the methods to screen indigenous Greek black pigs. The high number of viruses found in these animals allowed for the evaluation of numerous detection methods. Since porcine endogenous retroviruses (PERVs) type A and B are integrated in the genome of all pigs, but PERV-C is not, the animals were screened for PERV-C and PERV-A/C. Our detection methods were sensitive and detected PCMV/PRV, PLHV-1, PLHV-1, PLHV-3, PVC3 and PERV-C in most animals. PPV1, HEV3, PCV4 and PERV-A/C were not detected. These data are of great interest since the animals are healthy and resistant to diseases.

## 1. Introduction

The virome of pigs is not well studied [1]. The virome is the total amount of viruses in and on the pig body and also includes the endogenous retroviruses as well as the bacteriophages infecting bacteria present in the pig organisms. Most common in healthy pigs are picornaviruses followed by circoviruses, adenoviruses and parvoviruses [2]. In the case of diarrhea, the percentage of adenoviruses and circoviruses decreased and the percentage of anelloviruses and reoviruses increased [2]. In diarrhoeic faeces samples from 27 Chinese pigs, porcine bocavirus-2 (a parvovirus) was found in 59% of the animals, porcine bocavirus-4 (also a parvovirus) in 18%, Torque teno sus virus-2 (TTSuV-2) (an anellovirus) in 7%, porcine epidemic diarrhea virus (PEDV) (a coronavirus) in 70%, porcine stool associated circular virus (PoSCV) (circovirus-like) in 7%, sapovirus (a calicivirus) in 33%, sapelovirus (a picornavirus) in 48%, torovirus (a coronavirus) in 33%, posavirus-1 (a picornavirus) in 40%, porcine astrovirus in 74%, coronavirus in 7%, porcine enterovirus-9 (a picornavirus) in 85%, picobirnavirus (PBV) in 15% and kobuvirus (a picornavirus) in 44% of the animals [3]. These figures provide an insight into the large number of viruses in healthy and diseased pigs.

In a recent study in the United States, serum samples from healthy show pigs from the years 2018–2019 were analyzed by high-throughput sequencing to estimate the virome. Results demonstrated the presence of DNA viral families (*Parvoviridae*, *Circoviridae* and *Herpesviridae*) and RNA families (*Arteriviridae*, *Flaviviridae* and *Retroviridae*). Twenty-three viral species were identified. Among them were important swine pathogens including porcine reproductive and respiratory syndrome virus (PRRSV), atypical porcine pestivirus and porcine circovirus (PCV) [4]. The herpesvirus detected was PCMV/PRV, but only one contig. This underlines the fact that next-generation sequencing (NGS) can detect known viruses but has an extremely limited sensitivity. When 36 pooled porcine nasal swabs and blood serum samples collected from both sides of the Dutch-German border region were evaluated, 46 different viral species were detected using viral targeted sequence capture (TSC) compared to 40 viral species with a shotgun metagenomics approach [5]. In contrast, more sensitive methods such as PCR and real-time PCR can detect viruses even at a very low virus load [1].

Studies on the prevalence of porcine viruses were stimulated by the rapid development of xenotransplantation using pig cells, tissues and organs. Xenotransplantation is under development to alleviate the shortage of human donor organs for the treatment of organ failure. In recent years, remarkable survival times of pig xenotransplants in non-human primates were achieved. In fact, the first two pig hearts were transplanted into patients in Baltimore recently [6,7]. However, xenotransplantation may be associated with the transmission of porcine viruses, which may be zoonotic or xenozoonotic. Viruses are zoonotic when they can cause a disease in healthy humans such as the hepatitis E virus, genotype 3 (HEV3), which is common in pigs. Viruses are xenozoonotic when they do not induce a disease in healthy humans but affect the recipient when transmitted with a xenotransplant such as PCMV/PRV [8]. It was shown that the transmission of PCMV/PRV drastically reduced the survival time of pig xenotransplants in non-human primates [9,10]. PCMV/PRV was also transmitted to the first patient in Baltimore and probably contributed to his death [6,11]. To prevent the transmission of potentially zoonotic or xenozoonotic pig viruses, sensitive and specific detection methods should be developed and evaluated (for review see [12]). It became clear that for a successful detection of pig viruses, not only sensitive and specific detection methods (either PCR-based, cell-based or immunological methods) are required. An entire “detection system” including sample generation, sample preparation, sample origin, time of sampling as well as negative and positive controls is important [12]. 

**Figure 1:**
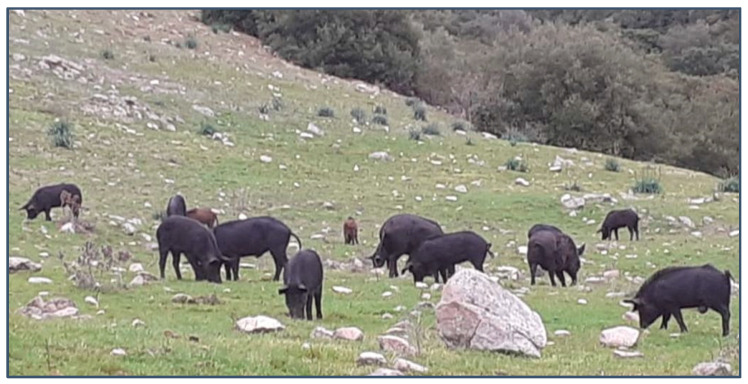
Indigenous Greek black pigs on a mountainous pasture in Greece (Photo: Vasileios Papatsiros).

Here, we analyzed another pig breed—the indigenous Greek black pigs (Figure 1)—using these methods. This breed is the only traditional indigenous pig breed reared in Greece. Most interestingly, it has its roots in ancient Greece. It is thought that these are the pigs from the Odyssey in the farm of Odysseus with his swineherd Eumaios [13].

Most Greek organic pig farms are located in mountainous or semi-mountainous areas, which is why they do not have a thick layer of fat like other types of pigs. They are resistant to weather conditions and diseases. Conventional pigs give birth to 12–14 piglets, whereas indigenous Greek black pigs give birth to a maximum of 7. A conventional pig is utilized at the age of 5 months and weight of 110 kg, while an indigenous Greek black pig is slaughtered at 7–10 months of age, reaching a carcass weight of about 60 kg [14,15]. The animals give delicious pork meat and in some farms are fed with olives. When the genetic diversity, based on microsatellite analysis, of the Greek black pig was investigated, its genetic uniqueness was demonstrated. Despite their low population size, they have a high degree of genetic variability, which will be useful for breeding programs aimed at maintaining the long-term survival of this ancient breed [14,15].

**Figure 2:**
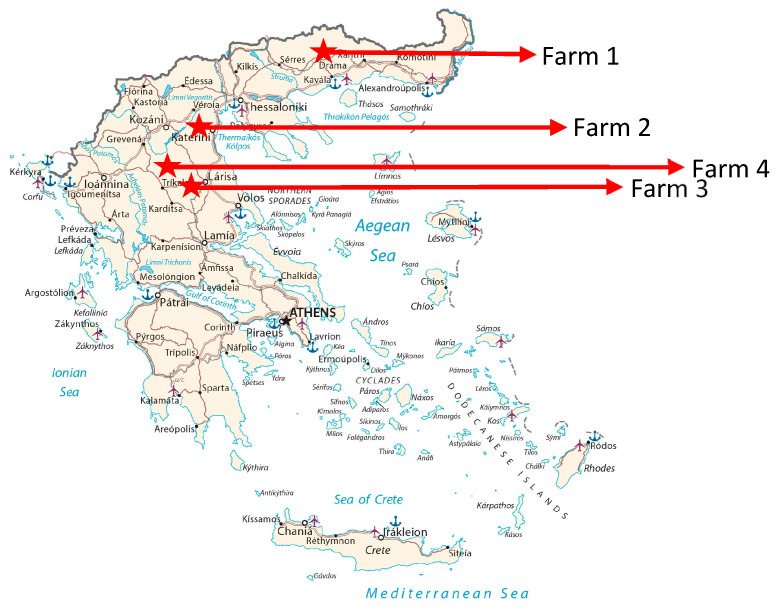
Localisation of the farms that supplied the indigenous Greek black pigs analyzed here.

Twenty-one animals from four farms in Greece (Figure 2) were analyzed using real-time PCR for PCMV/PRV, PCV2, PCV3, PCV4, PLHV-1, PLHV-2 and PLHV-3, as well as real-time RT-PCR for HEV3. For the detection of PERV-C and PERV-A/C, conventional PCRs were used. In addition, eleven animals from two farms were screened for antibodies against PCMV/PRV using a Western blot assay.

## 2. Materials and Methods

### 2.1. Animals and Tissues

Liver and some spleen tissues were obtained from animals in four different farms in Greece (Figure 2). Farm 1 is located near Drama, North Greece (four samples). Farm 2 (three samples), farm 3 (six samples) and farm 4 (eight samples) are all located near Trikala, Thessaly, Central Greece. In addition, sera were obtained from 11 animals from farms 1 and 4. The tissue samples were taken in slaughterhouses and the animals were healthy and suitable for human consumption. Their age ranged between 4 and 36 months.

### 2.2. DNA and RNA Isolation

DNA and RNA were isolated from the tissues according to the manufacturer’s instructions using the DNeasy Blood and Tissue kit and RNeasy kit (Qiagen, Hilden, Germany), respectively. DNA and RNA concentrations were determined using NanoDrop ND-1000 (Thermo Fisher Scientific Inc., Worcester, MA, USA).

### 2.3. Real-Time PCR for the Detection of DNA Viruses

Real-time PCRs were performed to detect PCMV/PRV, PLHV-1, PLHV-2, PLHV-3, PCV2, PCV3, PCV4 and PPV1 as described previously using specific primers and probes (Table 1) [16,17,18,19,20,21,22]. All protocols were performed using the SensiFAST Probe No-ROX kit (Meridian Bioscience, Cincinnati, OH, USA) in a reaction volume of 16 µL plus 4 µL (100 ng) of DNA template. All real-time PCRs were carried out as duplex PCRs that simultaneously indicate the gene of interest and porcine glyceraldehyde-3-phosphate-dehydrogenase (pGAPDH) as internal control for each sample. Real-time PCR reactions were carried out with a qTOWER3 G qPCR cycler (Analytik Jena, Jena, Germany) and the real-time PCR conditions as previously described [16].

### 2.4. Real-Time Reverse Transcriptase PCR for the Detection of HEV3

Real-time reverse transcriptase-PCR (real-time RT-PCR) as described by Jothikumar et al. [23] was carried out to detect hepatitis E virus, genotype 3 (HEV3). All real-time RT-PCR reactions were performed in a reaction volume of 16 µL using SensiFAST Probe No-ROX One-Step Kit (Meridian Bioscience, Cincinnati, OH, USA) plus 4 µL (100 ng) template RNA. The reaction was performed at the qTOWER^3^ G qPCR cycler (Analytik Jena, Jena, Germany). The temperature-time profile applied consists of a reverse transcriptase step of 30 min at 50 °C, followed by an activation step of 15 min at 95 °C and 45 cycles comprising a step of 10 s at 95 °C, followed by a step of 20 s at 55 °C and 15 s at 72 °C [24].

### 2.5. Conventional PCR for the Detection of PERVs

A conventional PCR was performed to determine the presence of PERV-C. PERV-C was detected using a set of primers with an amplicon length of 288 bp (described as PCR4 in [25]). It was carried out with AmpliTaq DNA Polymerase (Applied Biosystems, Waltham, MA, USA) and was set up with a Biometra TRIO cycler (Analytik Jena, Jena, Germany). The following temperature-time profile was used: 95 °C for 10 min (activation step), followed by 45 cycles at 95 °C for 15 s (denaturation), 55 °C for 30 s (annealing) and 72 °C for 30 s (extension) and a final extension at 72 °C for 5 min.

A conventional PCR to determine the presence of human-tropic PERV-A/C was set up using specific primer pairs (Table 1) [26]. The PERV-A/C long primer mix detects an amplicon of 1266 bp length. It was carried out with AmpliTaq DNA Polymerase (Applied Biosystems, Waltham, MA, USA) and was set up with a Biometra TRIO cycler (Analytik Jena, Jena, Germany). The following temperature-time profile was used: 95 °C for 10 min (activation step), followed by 45 cycles at 95 °C for 15 s (denaturation), 55 °C for 30 s (annealing), 72 °C for 90 s (extension) and a final single cycle at 72 °C for 5 min.

### 2.6. Determination of the Sensitivity

The sensitivity of real-time PCRs for the detection of various viruses tested in this study was determined using gene blocks (Integrated DNA Technologies, IDT, Coralville, IA, USA) and were used as described [24]. The gene blocks (gBlocks) comprised the virus-specific oligosequences corresponding to the primer and probe, which are separated by spacers. The spacers are non-functional oligosequences of eight to fifteen base pairs that are used as placeholder sequences. In addition to the viral gene blocks, we used also a gene block containing the primer and probe sequences of the porcine GAPDH. Full sequences and characterization of the gBlocks are given in [24]. Different copy numbers (10^1^ to 10^9^) of gene blocks comprising of various virus sequences were detected with respective primers and probes (Table 1) [24]. It was performed using the SensiFAST Probe No-ROX kit (Meridian Bioscience, Cincinnati, OH, USA) in a reaction volume of 16 µL plus 4 µL (100 ng) of DNA template.

**Table 1:** Oligonucleotides for the primers and probes used in this study.

Virus	Primer/Probe	Sequence 5′–3′	Reference
HEV3	JVHEV3-Fwd	GGT GGT TTC TGG GGT GAC	Jothikumar et al., 2006 [23]
	JVHEV3-Rev	AGG GGT TGG TTG GAT GAA	
	JVHEV3-Probe	6FAM-TGA TTC TCA GCC CTT CGC-BHQ	
PCMV/PRV	PCMV-Fwd	ACT TCG TCG CAG CTC ATC TGA	Mueller et al., 2002 [17]
	PCMV-Rev	GTT CTG GGA TTC CGA GGT TG	
	PCMV-Probe	6FAM-CAG GGC GGC GGT CGA GCT C-BHQ	
PLHV-1	PLHV-1 (1125)-Fwd	CTC ACC TCC AAA TAC AGC GA	Chmielewicz et al., 2003 [18]
	PLHV-1 (1125)-Rev	GCT TGA ATC GTG TGT TCC ATA G	
	PLHV-1 (1125)-Probe	6FAM-CTG GTC TAC TGA ATC GCC GCT AAC AG-TAMR	
PLHV-2	PLHV-2 (1155)-Fwd	GTC ACC TGC AAA TAC ACA GG	Chmielewicz et al., 2003 [18]
	PLHV-2 (1155)-Rev	GGC TTG AAT CGT ATG TTC CAT AT	
	PLHV-2 (1155)-Probe	6FAM-CTG GTC TAC TGA AGC GCT GCC AAT AG-TAMRA	
PLVH-3	PLHV-3 (210s)-Fwd	AAC AGC GCC AGA AAA AAA GG	McMahon et al., 2006 [19]
	PLHV-3 (210as)-Rev	GGA AAG GTA GAA GGT GAA CCA TAA AA	
	PLHV-3 (210)-Probe	6-FAM CCA AAG AGG AAA ATC-MGB	
PCV2	PCV2 (F2020)-Fwd	CTG AGT CTT TTT TAT CAC TTC GTA ATG GT	Chen et al., 2021 [20]
	PCV2 (F2020)-Rev	ACT GCG TTC GAA AAC AGT ATA TAC GA	
	PCV2 (F2020)-Probe	6FAM-TTA AGT GGG GGG TCT TTA AGA TTA AAT TCT CTG AAT TGT-BHQ2	
PCV3	PCV3-Fwd	AGT GCT CCC CAT TGA ACG	Palinski et al., 2017 [21]
	PCV3-Rev	ACA CAG CCG TTA CTT CAC	
	PCV3-Probe	6FAM-ACC CCA TGG CTC AAC ACA TAT GAC C-BHQ1	
PCV4	PCV4 (F2020)-Fwd	ATT ATT AAA CAG ACT TTA TTT GTG TCA TCA CTT	Chen et al., 2021 [20]
	PCV4 (F2020)-Rev	ACA GGG ATA ATG CGT AGT GAT CAC T	
	PCV4 (F2020)-Probe	6FAM-ATA CTA CAC TTG ATC TTA GCC AAA AGG CTC GTT GA-BHQ1	
PPV1	PPV1-Fwd	CAG AAT CAG CAA CCT CAC CA	Opriessnig et al., 2011 [22]
	PPV1-Rev	GCT GCT GGT GTG TAT GGA AG	
	PPV1-Probe	6FAM-TGC AAG CTT/ZEN/AAT GGT CGC ACT AGA CA-BHQ1	
pGAPDH	pGAPDH-Fwd	ACA TGG CCT CCA AGG AGT AAG A	Duvigneau et al., 2005 [25]
	pGAPDH-Rev	GAT CGA GTT GGG GCT GTG ACT	
	pGAPDH-Probe	HEX-CCA CCA ACC CCA GCA AGA G-BHQ1	
PERV-C	PERV-envC-Fwd	GAT TAG AAC TGG AAG CCC CAA GTG CTC T	Kaulitz et al., 2013 [26]
	PERV-envC-Rev	TCT GAT CCA GAA GTT ATG TTA GAG GAT GGT	
PERV-A/C	PERV-A env VRBF-Fwd	CCT ACC AGT TAT AAT CAA TTT AAT TAT GGC	Wood et al., 2004 [27]
	PERV-C env TMR-Rev	CTC AAA CCA CCC TTG AGT AGT TTC C	

Fwd = forward primer, Rev = reverse primer.

### 2.7. Western Blot to Detect Antibodies against PCMV/PRV

Western blotting was performed as described previously in detail using the recombinant R2 fragment of the gB protein of PCMV/PRV [28,29,30]. The sera were tested at a dilution of 1:150.

## 3. Results

### 3.1. Sensitivity of the Assays

To determine the sensitivity of the real-time PCRs, gene blocks were used as described [24]. The sensitivity of our real-time PCRs was determined (Supplementary Figure S1) and compared with the sensitivity of previously published real-time PCRs (Table 2). The sensitivity of the real-time PCRs ranged between one and ten copies (Table 2).

### 3.2. Screening for Herpesviruses: PCMV/PRV, PLHV-1, PLHV-2, PLHV-3

When the animals were tested for these herpesviruses, 16 of 21 animals were positive for PCMV/PRV in liver tissues, 12 animals were positive for PLHV-1, 15 animals were positive for PLHV-2 and all were positive for PLHV-3 (Table 3). Similar results were obtained when spleen tissue was tested (Table 4).

### 3.3. Screening for Circoviruses and PPV1

All animals were positive for PCV2 and 6 of the 21 were positive for PCV3 (Table 3). Farm 4 was the only one free of PCV3. We did not test for PCV1 because PCV1 was found non-pathogenic in pigs [31]. None of the animals were PCV4 and PPV1 positive (Table 3).

### 3.4. Screening for RNA Viruses: HEV3

All animals were free from HEV3 (Table 3).

### 3.5. Screening for PERVs

**Figure 3:**
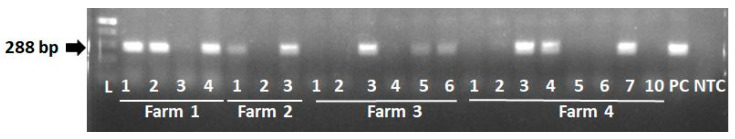
Results of the PCR testing for PERV-C. Animals from all farms were tested, DNA was from the liver. PC, positive control; NTC, negative control.

Since all pigs harbor PERV-A and PERV-B in their genome, we tested only for PERV-C using a primer pair described previously (PCR4 in [26]). Eleven of the twenty-one animals were positive for PERV-C using this PCR (Table 3, Figure 3). When the PERV-C-positive animals were tested for PERV-A/C, this recombinant virus was not found (Table 3).

**Table 2:** Sensitivity of different PCR-based methods detecting pig viruses.

Virus	Method	Sensitivity (Copy Number Per 100 ng DNA)	Sensitivity R^2^	Reference
PCMV/PRV	conventional PCR	15 copies		Morozov et al., 2016 [32]
	nested PCR	5 copies		
	real-time PCR	2 copies		
	real-time PCR	20 copies		Mueller et al., 2002 [17]
	real-time PCR	10 copies	0.9964	this manuscript
HEV3	real-time RT-PCR	10 copies		Jothikumar et al., 2006 [23]
	real-time RT-PCR	150–200 copies		Morozov et al., 2015 [33]
	real-time RT-PCR	10 copies	0.9962	this manuscript
PCV2	multiplex	101 copies/µL		Zhou et al., 2022 [34]
	real-time PCR	1 copy	0.9935	this manuscript
PCV3	real-time PCR	10 copies	0.9906	this manuscript
PCV4	real-time PCR	100 copies	0.9906	this manuscript
PLHV-1	real-time PCR	20 copies		Chmielewicz et al., 2003 [18]
	real-time PCR	1 copy	0.9964	this manuscript
PLHV2	real-time PCR	20 copies		Chmielewicz et al., 2003 [18]
	real-time PCR	1 copy	0.9953	this manuscript
PLHV3	real-time PCR	1 copy	0.9983	this manuscript
PPV1	real-time PCR	10 copies	0.9961	this manuscript

### 3.6. Western Blot Assay to Detect Antibodies against PCMV/PRV

**Figure 4:**
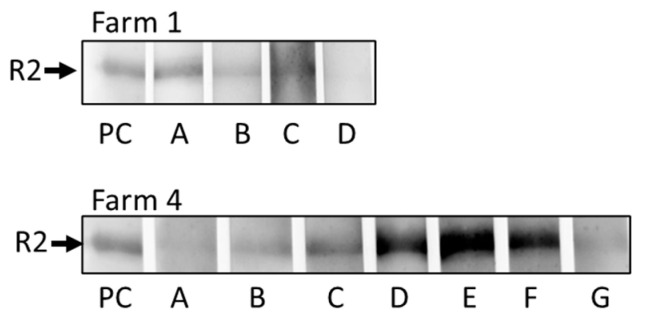
Results of the Western blot analysis to detect antibodies against PCMV/PRV. Animals A, B, C and D from farm 1 and animals A–G from farm 4 were tested. PC, positive control. Exposition time: 9 ms.

A Western blot analysis of sera from animals from farms 1 and 4 was performed using the recombinant C-terminal fragment of gB of PCMV/PRV [28]. The tested animals were not the same as the animals tested using the PCR, but in the same age (four animals from farm 1, aged eight–nine months; seven animals from farm 4, aged ten–eleven months). All sera reacted positive (Figure 4), however, with strong differences in the extent of the band. The serum from animal D from farm 1 seems to be negative in Figure 4, however, after using a longer exposition time (2 s instead of 9 milliseconds), these animals were found positive. Our assay is specific: negative sera were negative at higher exposition times (10 s). Furthermore, using this Western blot assay, similar results were obtained compared with an ELISA using synthetic peptides [35].

**Table 3:** Screening for pig viruses in liver of indigenous Greek black pigs (mean ct values).

Animal	Age (Months)	PCMV/PRV	PLHV-1	PLHV-2	PLHV-3	PPV1	PCV2	PCV3	PCV4	HEV3	PERV-C	PERV-A/C
		Real-Time PCR	Real-Time PCR	Real-Time PCR	Real-Time PCR	Real-Time PCR	Real-Time PCR	Real-Time PCR	Real-Time PCR	Real-Time RT-PCR	PCR	PCR
	Farm 1											
1	8–9	n.d.	33.75	33.31	28.49	n.d.	31.1	n.d.	n.d.	n.d.	+	−
2	8–9	34.31	n.d.	28.74	28.09	n.d.	31.35	32.85	n.d.	n.d.	+	−
3	8–9	n.d.	n.d.	27.33	34.24	n.d.	30.35	n.d.	n.d.	n.d.	−	−
4	8–9	33.49	n.d.	27.55	26.72	n.d.	27.58	34.02	n.d.	n.d.	+	−
	Farm 2											
1	11–12	n.d.	33.51	n.d.	29.91	n.d.	30.12	n.d.	n.d.	n.d.	+	−
2	11–12	33.56	32.87	n.d.	22.74	n.d.	32.43	n.d.	n.d.	n.d.	−	−
3	11–12	34.92	32.33	30.57	33.8	n.d.	34.52	25.17	n.d.	n.d.	+	−
	Farm 3											
1	4	33.44	n.d.	31.15	22.86	n.d.	18.66	29.69	n.d.	n.d.	−	−
2	36	35.32	n.d.	31.98	32.19	n.d.	33.37	29.45	n.d.	n.d.	−	−
3	4	32.00	n.d.	32.34	27.27	n.d.	32.8	28.02	n.d.	n.d.	+	−
4	4	31.94	28.99	27.04	36.44	n.d.	23.54	n.d.	n.d.	n.d.	−	−
5	5	n.d.	33.46	n.d.	31.8	n.d.	34.51	n.d.	n.d.	n.d.	+	−
6	5	n.d.	32.44	n.d.	34.33	n.d.	34.58	n.d.	n.d.	n.d.	+	−
	Farm 4											
1	10–11	29.94	33.98	15.69	24.24	n.d.	28.99	n.d.	n.d.	n.d.	−	−
2	10–11	29.8	28.23	25.58	24.54	n.d.	29.98	n.d.	n.d.	n.d.	−	−
3	10–11	29.42	30.22	n.d.	31.52	n.d.	21.69	n.d.	n.d.	n.d.	+	−
4	10–11	32.60	n.d.	29.88	27.26	n.d.	28.62	n.d.	n.d.	n.d.	+	−
5	10–11	32.47	n.d.	28.43	20.98	n.d.	31.14	n.d.	n.d.	n.d.	−	−
6	10–11	30.21	31.38	28.85	22.51	n.d.	26.1	n.d.	n.d.	n.d.	−	−
7	10–11	31.41	29.74	n.d.	31.79	n.d.	23.02	n.d.	n.d.	n.d.	+	−
8	10–11	31.70	n.d.	29.83	21.95	n.d.	26.03	n.d.	n.d.	n.d.	−	−

n.d., not detected; +, positive PCR result; −, negative PCR result.

**Table 4:** Comparison of the PCMV virus load in spleen and liver of four pigs in Farm 1.

Animal	Organ	PCMV	pGAPDH
1	spleen	n.d.	19.10
	liver	n.d.	19.72
2	spleen	31.34	18.58
	liver	34.31	19.41
3	spleen	n.d.	19.57
	liver	n.d.	19.89
4	spleen	32.32	20.00
	liver	33.49	19.17

n.d., not detected.

## 4. Discussion

In order to evaluate the potency of our improved detection methods developed for the effective screening of viruses potentially posing a risk for xenotransplantation, indigenous Greek black pigs were thoroughly screened. They were first tested for PCMV/PRV, which had been shown to reduce the survival time of pig transplants in non-human primates significantly [9,10]. PCMV/PRV was also transmitted in the first transplantation of a pig heart into a patient in Baltimore [6,11]. Since the symptoms in baboons with PCMV/PRV-positive transplants are similar to the symptoms in the Baltimore patient, the virus obviously contributed to the death of the patient. The real-time PCR developed by Mueller et al. [17] was modified and performed as a duplex real-time PCR detecting simultaneously porcine GAPDH [30]. Furthermore, gene blocks comprising the virus-specific oligosequences corresponding to the primers and probes were used as positive control and for the standard curves (Supplementary Figure S1). Using this real-time PCR, we detected 16 positive animals out of 21 (76%). In farm 4, all animals were infected, and it was in this farm where the animals with the highest virus load (ct values around 29) were found.

When sera from animals from farms 1 and 4 were analyzed in a Western blot using a recombinant C-terminal fragment of gB of PCMV/PRV, all tested animals were reacting positive (Figure 4). Some animals had a very faint reaction, e.g., animal D from farm 1 and animals A and G from farm 4. Animals D, E and F from farm 4 had a very strong reaction. The result is comparable with a Western blot testing of animals from a German slaughterhouse [28]. The R2 fragment was shown immunodominant in the gB protein [28] and gave similar results when compared with an ELISA using synthetic peptides corresponding to the R2 sequence [35,36]. Our Western blot assay was used repeatedly to determine the antibody response in different pig breeds [29,30].

When we started the investigation, we did expect a very low number of viruses due to the seclusion of the farms. However, the opposite was observed. This was a great advantage for our investigation because the detection methods can only be tested if viruses are present. Despite the high number of detected viruses, the animals were healthy and suitable for human consumption (the samples were collected from the slaughterhouse).

PLHV-3 was found in all tested indigenous Greek black pigs. This is a similar prevalence compared with other investigations. When 5 pigs in 22 farms in Ireland were screened for PLHV, every farm harbored animals infected with PLHV-1 (55%), and 82% of the farms scored positive for the presence of PLHV-2 and PLHV-3, respectively [19]. PLHV-1 was the most prevalent of the three species, followed by PLHV-3 and PLHV-2. Coinfections with two or even three viruses were reported. Despite the high prevalence of these viruses, until now, no association between PLHVs and any pig diseases had been described [37]. However, we recently described the finding of PLHV-3 in pigs with dippity pig syndrome (DPS) [16] and in Greek pigs with erythema multiforme [38]. Whether porcine lymphotropic herpesviruses, especially PLHV-3, pose a risk for xenotransplantation is unclear. The transmission of PCMV/PRV to the progeny can easily be prevented by caesarean section, which is not the case with PLHV. In one study, piglets obtained by somatic cell nuclear transfer (SCNT) and derived via caesarean section were screened using real-time PCR methods. PLHV-3 was detected in five of nine piglets and PLHV-2 in three of nine piglets [39]. In a study transplanting pig kidneys and hearts into immunosuppressed baboons, all donor pigs carried PCMV/PRV and 55% of them carried PLHV. PCMV was detected in all baboon recipients, but PLHV was not transmitted [40]. PLHV was also not transmitted to baboons through the hearts of all eight genetically modified pigs used for orthotopic pig heart transplantation which were all positive for PLHV-3 [10]. As mentioned, PLHV-3 was also found in pigs suffering from DPS [16] and from erythema multiforme [38]. However, it remains unclear whether the virus is involved in the corresponding pathogenesis.

Whereas all animals were positive for PCV2, only six animals were positive for PCV3 (Table 3). PCV2 causes porcine circovirus-associated diseases (PCVAD) including subclinical infection (PCV-2-SI), systemic (PCV-2-SD) and reproductive (PCV-2-RD) diseases and porcine dermatitis and nephropathy syndrome (PDNS) [29,41]. PCV2 was originally identified as the causative agent of post-weaning multisystemic wasting syndrome (PMWS) and the respiratory form of PCV2 has been classified as PCV2-associated respiratory disease or PCV2-lung disease (PCV2-LD) [42]. The situation with PCV3, which was also associated with pig diseases, was not clear from the beginning and it was thought that co-infections with other viruses were the reason for these diseases [43]. PCV3 was found in tissues of animals displaying PDNS and reproductive disorders [21]. However, it is clear that cloned PCV3 can induce disease in specified pathogen-free (SPF) pigs [44,45]. Therefore, it is interesting that there are pigs infected with PCV3 without any clinical signs, suggesting that some pig breeds have also a genetic resilience protecting them from the pathogenic properties of PCV3.

PCV4 was described for the first time in China in 2019 [46]. Recently, the first detection of PCV4 in Europe was reported in Spain and Italy [47]. Notably, the prevalence of PCV4 was higher in wild boars compared with commercial pigs. The fact that the indigenous Greek black pigs are free from PCV4 demonstrates that the virus has not penetrated remote Greek regions.

PPV1 causes infectious infertility [48]. It is associated with abortions in pigs and is considered as a possible trigger for the development of systemic disease in PCV2-infected pigs [49]. Although this virus is ubiquitous among pigs throughout the world, all indigenous Greek black pigs were free of PPV1 (Table 3). We did test only for PPV-1 because other than PPV1, pathogenicity of any of the other PPVs has not yet been conclusively demonstrated and it is unclear whether they can be transmitted to humans [49].

We did not test for classical swine fever virus (CSFV), African swine fever (ASFV), pseudorabies virus (also called Aujeszky virus or suide herpesvirus 1) and influenza virus because these viruses can be tested by veterinary laboratories and there was no need to establish these detection methods.

At present, HEV3 is the only virus with well-known zoonotic potential [50,51]. All indigenous Greek black pigs were free of HEV3 (Table 3).

Whereas PERV-A and PERV-B are present in the genome of all pigs, PERV-C is not. Eleven of the twenty-one tested indigenous Greek black pigs (52%) carried PERV-C in their genome. A low prevalence of PERV-C was found in some farms in the USA (6% up to 41%) [52] and in Chinese miniature pigs (30%) [53,54]. The presence of PERV-C opens the opportunity of a recombination with PERV-A. The resulting recombinant PERV-A/C was characterized by the ability to infect human cells with a high replication rate [55]. PERV-A/C were never found in the germ line, supporting the fact that PERVs are active in living animals and can integrate de novo and recombine [56]. All indigenous Greek black pigs tested were negative for PERV-A/C (Table 3).

The fact that in all indigenous Greek black pigs so many viruses were found even though the animals were healthy, since the samples were taken at the slaughterhouse, is of great interest. It suggests that the animals possibly have a natural resilience to virus infections due to the fact that they express many antiviral restriction factors which protect them. The situation seems to be similar to that of bats. Bats are recognized as important reservoirs of viruses deadly to other mammals, however, these viral infections are typically nonpathogenic in bats [57]. For example, bats possess more tetherin genes—an antiviral protein which prevents viral particles from escaping their host cell—than other mammals. Furthermore, some bats encode structurally unique tetherins [58]. Another restriction factor is that tripartite motif-containing protein 5 (TRIM5) was found in multiple copies in bats, and TRIM22 was often found duplicated in some bat species, an evolutionary phenomenon not yet observed in any other lineages of mammals [59]. Other bat species possess the largest and most diverse array of APOBEC3 genes identified in any mammal reported to date [60]. On the other hand, an excellent immune system may be the reason for the resilience of bats and all indigenous Greek black pigs [61]. It is possible that genetic markers could be associated with resistance to infectious diseases. Studies on Italian large white pigs, wild boars and local breeds indicate that the frequency of the resistance-associated alleles for four polymorphisms was usually higher in local pig breeds, indirectly supporting a higher rusticity of autochthonous breeds than in commercial populations [62]. Further research on the resistance of indigenous Greek black pigs to various viruses is required and will lead to important results as seen in the case of bats. It is clear that donor pigs used for clinical xenotransplantation should be free of potentially zoonotic or xenozoonotic viruses.

In a study on indigenous Greek black pigs, it was shown that this breed can be the reservoir of interesting genetic variants. In these animals a novel allele in the melanocortin 1 receptor (MC1R) gene was identified that was not previously reported in any other pig populations [63]. The novel allele leads to the production of different pigmentation. It was also shown that indigenous Greek black pigs experienced genetic admixture from two sources: wild boars and cosmopolitan breeds. This situation might raise concerns for the genetic integrity of this animal genetic resource, but on the other hand it may contribute to within-population genetic variability, therefore reducing the problem of inbreeding of the small population.

## 5. Conclusions

Indigenous Greek black pigs were tested for 11 porcine viruses which have relevance for the virus safety of xenotransplantation. The analyses of the sensitivity of the detection methods and the fact that all viruses except for PPV1, PCV4, HEV3 and PERV-A/C were detected, indicates the high sensitivity of the methods. Even though many viruses were detected, the animals were healthy, suggesting that they express active antiviral restriction factors. Further studies are needed to systematically investigate and understand the antiviral resistance of Greek black pigs against common pig viruses.

## Data Availability

All data supporting reported results can be found in the manuscript and the Supplementary Materials.

## Supplementary Materials

The following supporting information can be downloaded at: https://www.mdpi.com/article/10.3390/microorganisms12020315/s1, Figure S1: Results of the real-time PCRs using gene blocks: Standard curves indicating the sensitivity.
