# Supplementary figures and images for: Screening for Viruses in Indigenous Greek Black Pigs
Source: Microorganisms. 2024 Feb 2;12(2):315. doi: 10.3390/microorganisms12020315 (PMC10893322; doi:10.3390/microorganisms12020315)

Supplementary Figure S1

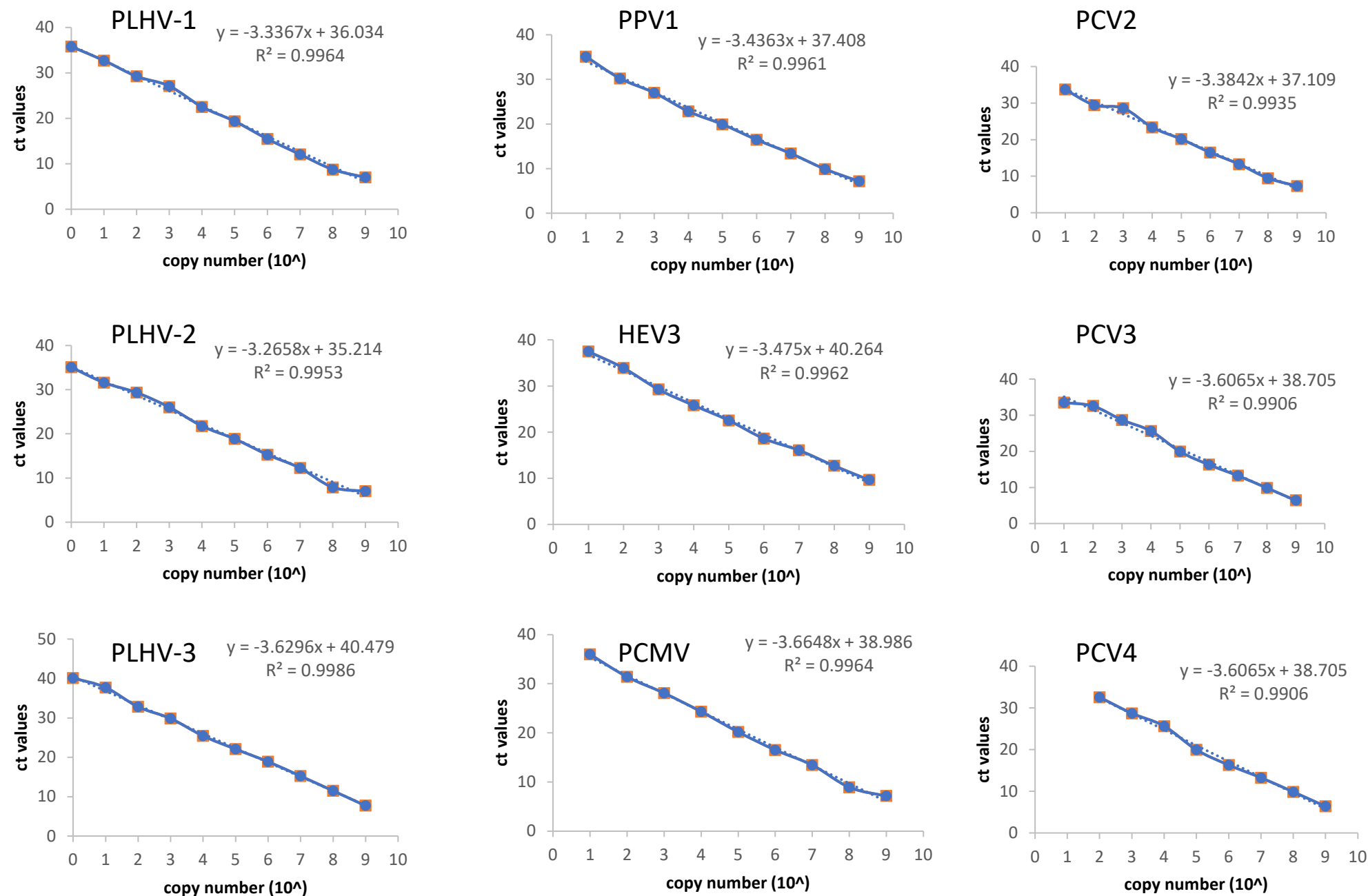

Supplement: Supplementary file 1 [file microorganisms-12-00315-s001.zip › microorganisms-2770856-supplementary.pdf]
